# Developing strategies to attract, retain and support midwives in rural fragile settings: participatory workshops with health system stakeholders in Ituri Province, Democratic Republic of Congo

**DOI:** 10.1186/s12961-020-00631-8

**Published:** 2020-11-04

**Authors:** Amuda Baba, Tim Martineau, Sally Theobald, Paluku Sabuni, Marie Muziakukwa Nobabo, Ajaruva Alitimango, John Kisembo Katabuka, Joanna Raven

**Affiliations:** 1 Institut Panafricain de Santé Communautaire, Aru, Democratic Republic of Congo; 2 Department of International Health, Liverpool School of Tropical Medicine, Liverpool, United Kingdom; 3 Université Officielle de Rwenzori, Country Director of the Leprosy Mission, Kinshasa, Democratic Republic of Congo

**Keywords:** strategies, participatory workshop, attraction, retention, midwives, rural, Abbreviations, DHMT District Health Management Team, NGO Non-governmental organisation, SBA skilled birth attendant, SDG Sustainable Development Goal, TBAs Traditional birth attendants

## Abstract

**Background:**

Midwifery plays a vital role in the quality of care as well as rapid and sustained reductions in maternal and newborn mortality. Like most other sub-Saharan African countries, the Democratic Republic of Congo experiences shortages and inequitable distribution of health workers, particularly in rural areas and fragile settings. The aim of this study was to identify strategies that can help to attract, support and retain midwives in the fragile and rural Ituri province.

**Methods:**

A qualitative participatory research design, through a workshop methodology, was used in this study. Participatory workshops were held in Bunia, Aru and Adja health districts in Ituri Province with provincial, district and facility managers, midwives and nurses, and non-governmental organisation, church medical coordination and nursing school representatives. In these workshops, data on the availability and distribution of midwives as well as their experiences in providing midwifery services were presented and discussed, followed by the development of strategies to attract, retain and support midwives. The workshops were digitally recorded, transcribed and thematically analysed using NVivo 12.

**Results:**

The study revealed that participants acknowledged that most of the policies in relation to rural attraction and retention of health workers were not implemented, whilst a few have been partially put in place. Key strategies embedded in the realities of the rural fragile Ituri province were proposed, including organising midwifery training in nursing schools located in rural areas; recruiting students from rural areas; encouraging communities to use health services and thus generate more income; lobbying non-governmental organisations and churches to support the improvement of midwives’ living and working conditions; and integrating traditional birth attendants in health facilities. Contextual solutions were proposed to overcome challenges.

**Conclusion:**

Midwives are key skilled birth attendants managing maternal and newborn healthcare in rural areas. Ensuring their availability through effective attraction and retention strategies is essential in fragile and rural settings. This participatory approach through a workshop methodology that engages different stakeholders and builds on available data, can promote learning health systems and develop pragmatic strategies for the attraction and retention of health workers in fragile remote and rural settings.

## Background

Maternal and newborn healthcare remains a particular focus in the Sustainable Development Goals (SDG) [1, 2]. Nurses and midwives make up the majority of the health workforce and play key roles in maternal and newborn healthcare [3, 4]. Midwives are a critical skilled birth attendant (SBA) cadre associated with improved quality care and rapid and sustained reductions in maternal and newborn mortality [5]. Midwives should work within an enabling environment, following guidelines for maternal and newborn care, providing and tailoring care to the social and cultural needs of women living in the community [6]. However, the UN report on the progress of the SDGs shows that many countries are still facing the challenge of shortages of qualified health workers, i.e. fewer than 1 doctor per 1000 population and fewer than 4 nurses and midwives per 1000 population [7]. Most sub-Saharan countries are amongst those countries [8, 9] and, within countries, there are serious distribution inequities between urban and rural areas [10].

To address the inequitable distribution of the workforce, WHO recommended interventions that could help attract and retain health workers in rural areas. These interventions focus on four areas, namely (1) medical education, where the focus is on the quality of the students and education settings as well as different learning strategies reflecting rural contexts; (2) regulatory interventions, that is, enhancing the scope of practice, producing different types of health workers, bonding schemes and compulsory service; (3) appropriate financial incentives; and (4) personal and professional support, including working and living conditions, safe and supportive work environment, and career development programmes [11]. These strategies need to be tailored to each country’s context [11, 12].

The Democratic Republic of Congo, which has one of the highest maternal mortality ratios in the world (846 per 100,000 live births), is classified as a state of high fragility [13]. Thirty years of mismanagement and two decades of conflict and unrest in the country have left the Democratic Republic of Congo among one of the most underdeveloped countries in the world; it is ranked as the third weakest country in the world, after Afghanistan and Somalia [14, 15]. The Democratic Republic of Congo experiences poor governance, infrastructure and basic services, with serious implications for its population’s living conditions and health indicators [16–19]. The Democratic Republic of Congo faces a severe shortage of SBAs, with only 1.05 physicians, nurses and midwives per 1000 population in 2012, compared to the SDG index threshold of 4.45 per 1000 population. The shortage of midwives in the Democratic Republic of Congo is particularly striking, with 0.06 midwives per 1000 population in 2015 and shortages, especially in rural areas [20–22], compared to neighbouring countries such as the Central African Republic with 0.114, Uganda with 0.200, Republic of Congo with 0.291, Tanzania with 0.435 and Zambia with 0.876 midwives per 1000 population (2012 figures) [23] More details about the midwifery profession in the Democratic Republic of Congo are given in Box 1.

Since 1999, the Ituri province (the study setting) has endured sustained socio-political crises and wars. Ongoing ethnic clashes and the recent Ebola outbreak make this province particularly fragile. Ituri, like some other provinces, faces challenges in attracting and retaining midwives in rural districts and has a high maternal mortality rate far beyond the national average and other rural provinces in the Democratic Republic of Congo [20, 24] (a situation confirmed by a personal communication with the Ituri Provincial Health Office). A recent study highlights the availability and distribution of midwives, nurses and doctors in Ituri province and the associated challenges (Box 2). Understanding midwives’ experiences in providing services in Ituri province is critical to addressing attraction and retention issues. A recent study documented these experiences, including the coping mechanisms deployed by midwives who demonstrate bravery and resilience as they navigate the interface position between under-resourced health systems and poor marginalised communities (Box 3).

The Ministry of Public Health developed strategies to attract and retain different categories of health workers in rural Democratic Republic of Congo such as improving living and working conditions, increasing financial incentives, registration of health workers, and introducing rural placement allowances (Box 4) [20, 21]. Many of these strategies are either not implemented or only poorly implemented, with serious implications on shortages of health workers, especially of midwives and rural areas, including in Ituri Province [20, 21, 25].

This study seeks to answer the question of, how can we better attract, support and retain midwives in the fragile and rural Ituri province of the Democratic Republic of Congo? This study engaged with different stakeholders in Ituri Province through a participatory approach to review the current midwifery staffing situation in order to identify feasible and context specific strategies that can help attract, support and retain midwives in the fragile and rural Ituri province.

## Methods

### Design

The qualitative participatory research design, through a workshop methodology [26], was used to develop appropriate contextual strategies to attract, support and retain midwives in rural and fragile Ituri. Participatory qualitative research enables local people to analyse, share and enhance their knowledge of life and condition and to plan, prioritise, act, monitor and evaluate [27]. Central to participatory methods is creating an empowering environment that places participants at the centre of the research and the facilitators/researchers as learners and enablers [28, 29]. It requires the establishment of credible and trusting relationships between researchers, individuals, groups and communities [30]. We used workshop methodology (see data collection section for details of the workshop) as it brings people together – as in this case, a wide range of health systems stakeholders – to learn, acquire new knowledge, perform creative problem-solving or innovate in relation to a specific issue, in this case, the attraction, retention and support of midwives. Furthermore, this methodology is specifically designed to produce reliable and valid data about the issue under study using group interaction [26, 31]. The workshop methodology uses a collaborative approach, where the researchers and participants work together, with the researchers facilitating inputs and discussion from all participants, ensuring that the strategies that are developed are grounded in the reality of Ituri province, are feasible to implement and will have impact [32]. The authors developed relationships with the participants as part of previous research (Baba et al., Unpublished data) [33], which helped generate collaborative and productive workshops.

### Setting

The research was carried out in three districts in Ituri Province, one of the 26 provinces of the Democratic Republic of Congo, a large province located in North-eastern Democratic Republic of Congo with a population of 5.4 million inhabitants. Three health districts were purposively selected by the research team in collaboration with the Provincial Health Office. The following districts were selected: Bunia district, where the Provincial capital is found and where all the facilities are in the urban area, including the district health office (Bunia); the peri-urban district of Aru, where there is a concentration of people, with some facilities in remote areas (Aru); and the rural district of Adja, where all facilities are in rural and remote areas [25]. IPASC (Pan African Institute of Community Health), a faith-based organisation where some of the authors work, organises community health-related interventions in the three health districts, which means we already understand the context and have relationships with the district health management. These relationships facilitated access to these districts, which are also relatively secure.

### Sampling and recruitment of participants

Purposive sampling was used for this study. The purposive sampling technique is the deliberate choice of participants based on features or characteristics that will enable a detailed understanding of the topic. The researcher sets out to find people who can and are willing to provide the information by virtue of knowledge or experience [34–36]. A range of decision-makers, managers and midwives were purposively selected based on their involvement in managing and supporting midwives or their experiences of being managed and providing services. Table 1 provides details of the different participants, including the reasons for their selection. In the previous study we had asked the midwives and head nurses if we could contact them again for participation in this study. We then contacted them directly by phone to recruit them to the workshops. Participants from the Provincial Health Office, Provincial Reproductive Health Coordination, District Health Management Team (DHMT), church medical coordination, non-governmental organisations (NGOs) and nursing schools were invited to the workshops through invitation letters and a participant information sheet. At the start of the workshop, the participant information sheets were shared again, discussed and written informed consent was obtained from each participant. In total, we recruited 49 participants to the three workshops, as described in Table 1 (15 in Bunia district, 19 in Aru district and 15 in Adja district).

**Table 1:** Participants of the workshops

Participant	Rationale for inclusion	Bunia	Aru	Adja	Total
Provincial Health Office Staff	Responsible for recruiting and deploying health workers within the Province, including midwives	3 (3 M)	0	0	3
Provincial Reproductive Health Coordination staff (PRHC)	Oversee in-service training of midwifery cadres within the province	2 (1 M; 1W)	0	0	2
DHMT members	Responsible for human resources for health management at district level	1 (W)	3 (3 M)	3 (3 M)	7
Church medical coordination staff	Church owns many health facilities in the province and employs midwives	0	1 (M)	1 (M)	2
NGOs focusing on maternal health	Collaborate with PRHC to provide in-service training for midwives and improve their working conditions	1 (W)	1 (W)	2 (1W; 1M)	4
Head nurses	Direct managers of midwives	3 (1 M; 2W)	7 (2 M; 5W)	3 (3 M)	13
Nursing school staff	Responsible for the training of midwives	0	2 (1 M; 1W)	0	2
Midwives	Provide maternal healthcare services	5 (1 M; 4W)	5 (1 M; 4W)	6 (6W)	16
**Total**		**15**	**19**	**15**	**49**

*DHMT* District Health Management Team, *M* Man, *NGO* non-governmental organisation, *W* Woman, *PRHC* Provincial Reproductive Health Coordination

### Data collection

A workshop was held in each district in November 2019. They were facilitated by AB with support from three research assistants (AA, JK and MM) and were designed so that research data and local policies were presented and discussed and then strategies were developed. Each workshop included five steps.

Is step 1, the facilitators presented data from the study on midwives’ work experiences and challenges through time from initial professional choice to future career aspiration in rural Ituri Province, north-eastern Democratic Republic of Congo. Participants discussed these findings as wall as what they had learnt and what problems the results reveal about SBAs in Ituri province. In step 2, data on the availability and distribution of SBAs in Ituri province was presented. Participants again discussed this data, highlighting the main issues. In step 3, the current policies on attraction and retention of health workers in rural areas in the Democratic Republic of Congo were presented. In step 4, participants were divided into groups (Table 2) so that they could discuss in detail the research findings and the policies. These discussions were facilitated by the research team (AB, JK and MM) using a topic guide that focused on policy implementation and reasons why some policies are implemented and others are not. The group discussions were summarised and fed back to the plenary for further discussion. Finally, in step 5, the groups discussed new and existing strategies to promote the attraction, retention and support of midwives, and how they could be implemented in their districts. They discussed challenges related to each strategy and how they could be overcome. After completion, each group reported to the other groups for further comments and discussions.

**Table 2:** Attraction and retention strategy discussion groups by district and membership

Bunia workshop	Aru workshop	Adja Workshop
DHMT delegate, Provincial Health Office delegates, NGO delegate	DHMT delegates, NGO delegate, church medical coordination delegate, nursing schools’ delegates	DHMT delegates, NGO delegate, church medical coordination delegate, head nurses
Head nurses and midwives	Midwives	Midwives
	Head nurses	

*DHMT* District Health Management Team, *NGO* non-governmental organisation

All discussions were recorded, stored in a password-protected computer, transcribed verbatim and then translated into English by an external translator from the Teaching College of Bunia and crosschecked by another external translator.

### Data analysis

We used the thematic framework method to analyse the data from the workshops [37]. This method facilitates rigorous and transparent analysis and uses both deductive and inductive approaches [37–39]. A coding framework was developed by all authors based on themes emerging from the data and the study objectives. Using this framework, AB coded the transcripts and shared with co-authors (JR, ST) to check coherence and meaning. When there were discrepancies in coding, the issue was discussed with all authors until a consensus was reached. The coding framework was applied to transcripts of all workshops, charts were then developed for each theme, and these charts were used to describe the themes. NVIVO 12 was used to support the analysis.

### Ethics approval

Ethics approval for this study was granted by the Liverpool School of Tropical Medicine Ethics Committee (Research protocol 17–024) and the CRMD/Bunia (Centre de Recherche Multidisciplinaire pour le Développement) (018/2017). A rigorous informed consent process was followed, where all participants were given verbal and detailed written information about the nature and purpose of the research before taking part. Furthermore, participants were made aware of their right to decline answering questions and were assured that measures are in place to anonymise responses. All participants gave written consent. All data were anonymised.

## Results

The results are presented in two sections. The first section presents participants’ perceptions and experiences of the midwifery and maternal healthcare situation in Ituri province and the implementation of the current policies on attraction and retention of health workers in rural areas. The second section focuses on the strategies developed in the workshop.

### Midwifery, maternal healthcare situation and policy implementation in Ituri province

All participants reported that most of the elements in the policies are not implemented or are only implemented in some areas in Ituri province. The main reason given was that the policies are developed at central level and no plans for implementation were made. They also explained that no funds are provided to the provincial level to implement the polices.

“*As you may know, policies are developed at the national level, and our roles in the district health level is to implement. But, we cannot implement if there are no funds for the implementation.*” (DHMT, Man, Aru)

Many participants from all districts also provided examples of inequitable implementation of policies. The allowance that should be provided to all health workers working in remote rural areas is not being provided. This exacerbates the inequity as the risk allowance, which is an entitlement for all staff, is mainly provided to health workers working in urban areas and rarely reaches rural workers.

“*If only they implemented remote and rural placement allowances, things would have changed automatically. It is just on paper, and nothing is really done on that*” (Midwife, Woman, Bunia).

“*… most health workers working in urban areas receive their risk allowances compared to those in rural areas …*” (Head Nurse, Man, Adja)

Many participants in all districts explained that the Provincial Health Office has limited control over the recruitment and deployment of health workers, which may be partly attributed to the human resources for health information system being unreliable as it is not updated. This has resulted in inequitable distribution of health workers across the districts.

“*At the provincial level, normally they should promote recruitment of different health workers’ categories according to the staffing standards, but the way different health workers are being recruited does not help to cover the gaps at the health district level*” (Provincial Health Office, Man, Bunia)

The DHMT has the responsibility for controlling staffing in all facilities in the districts. However, in reality, there are many staff and surpluses in some facilities, mainly in urban areas, and shortages in facilities located in rural and remote areas.

“*At the health district level, they should also respect and control HRH* [human resources for health] *staffing in different health facilities, making sure that they do not have a plethora of qualified health workers in urban health areas, they should work on convincing qualified health workers to go to serve in rural areas.*” (Head nurse, Man, Aru)

All participants described a severe shortage of midwives throughout the province – in urban as well as rural areas – and that a key factor contributing to this shortage is that only three of the 17 schools of nursing in the province provide midwifery training, resulting in an insufficient number of graduates.

“*As you can realise, in different nursing schools for secondary level, they only had nursing programme, there was none on midwifery*” (DHMT, Male, Aru)

In addition, all participants explained that the findings from the previous studies show that the midwifery profession is not attractive in these districts. In particular, they stressed the heavy workload because of a shortage of midwives, difficult working conditions because of limited resources and ongoing conflict, and no provision of salary or rural remote allowance but occasional risk allowance.

Some midwives perceived that their profession, especially in rural areas, receives little attention from the government:

“*When I look at all challenges that are described in this study* [as described in the workshop]*, I find them being real realities. When you are a midwife, especially in rural areas, you face all those challenges. My concern is on the profession of midwives. They* [the government] *should really consider their profession seriously, especially as they deal more with women and babies’ lives.*” (Midwife, Woman, Aru)

### Strategies for attraction and retention of midwives in remote and rural areas

We used the WHO framework of recommendations to increase access to health workers in remote and rural areas through improved retention to present the strategies [11]. To begin this section, Table 3 provides a summary of the strategies, challenges with implementation and possible solutions that were proposed in the workshops. Each category of intervention is then discussed in-depth.

**Table 3:** Strategies to increase attraction and retention of midwives in remote and rural areas

Categories of intervention	Proposed strategies		Challenges	Possible solutions
	National	Local/district		
**Education**	Promotion of nursing schools organising midwifery in rural areas	Recruiting and training rural background students	Poverty Community ignorance Lack of children to be recruited Conflict generated from selection candidates	Community-based education sponsorship scheme for recruited students
**Regulatory**	Registration of rural-based midwives	Recruiting and integrating TBAs in facilities	No salary for TBAs Continuation of providing home delivery	Salary from user fees Local authority involvement to ban home delivery
**Financial incentives**	Salary of health workers from the central government Implementation of rural placement allowances	Increased salary from income generated from user fees	Lack of funding from the government Flat rates imposed by NGOs Poverty of the rural population	Difficult to overcome Increased local income generated from user fees
**Professional and personal support**	Better living conditions Safe and supportive working environment	Good relationship at the facility and with the community Good leadership at different levels (communities, facilities, DHMTs)	Lack of funding from the central government Unrest or insecurity	Community/church mobilisation to improve building conditions and houses for health workers District initiative on fund raising Lobbying to NGOs Difficult to overcome insecurity Strengthening supportive supervision and in-service training by church medical coordination and NGOs in the area
**Other local interventions**		Promoting interactions and contacts with students at nursing schools and colleges Promoting local marriage Recruiting and integrating TBAs in facilities	Church regulations Socio-cultural-related challenges No salary for TBAs Continuation of providing home deliveries by TBAs	Community mobilisation on the importance of education and midwifery TBAs salary from user fees Local authority involvement to ban home delivery

*DHMT* District Health Management Team, *NGO* non-governmental organisation

### Education strategies

Participants from rural Adja district proposed investment by the central government and promotion of nursing schools in rural areas to include midwifery training in rural areas.

“*… I think it is better that the government invests themselves in covering rural health districts with nursing schools, where they also integrate midwifery.*” (DHMT, Man, Adja)

Finances to create new nursing schools or to integrate midwifery in existing schools was identified as the main challenge by nursing schools’ delegates, DHMT delegates, NGO delegates and head nurses from Aru and Bunia districts. To overcome this challenge, participants from peri-urban Aru and rural Adja districts recommended that the government encourage churches to organise midwifery training in their existing schools of nursing or to establish new schools to cover rural areas.

“*… at this condition, the government should just encourage churches, with the big number of facilities they have, especially in rural areas, to organise nursing schools*” (Head Nurse, Man, Adja)

District level participants from the three districts recommended the selection and training of students from rural areas, who could be supported financially by their community, as this would encourage students to return to their rural communities when they complete their training.

“*If the local chief could contribute to send someone, a child from the area, who knows very well the area to study midwifery. When they will complete, they will most likely go back. For instance, there is someone who has just completed her midwifery training in one of the nursing schools, she said that she is from Yekia, and would like to go to serve at Yekia as a midwife.*” (DHMT, Man, Adja)

Challenges to implementing this strategy were around poverty, gender norms and creating community tensions. Poverty of families and communities and ongoing conflict hinders children’s inclusion in school education to a level that they cannot enrol on midwifery courses nor pay for midwifery training fees and other related costs. In addition, tensions in communities are sometimes created where some members are selected and others are not.

“*In relation to identifying local children to send them to train midwifery in nursing schools and colleges could be community ignorance on the importance of schooling their children, especially for some specific courses; there is poverty of the local community, conflict in the community as someone’s child can be selected, and those whose children are not selected might end up not being happy.*” (Midwife, Man, Bunia)

This is also influenced by limited focus placed on education by communities, which is a particular challenge for rural girls/women as there are perceptions that they should marry rather than go for education. This compounds the problem of the shortage of midwives in rural areas, as rural communities prefer to have female midwives.

“*You know that in Ituri, it maybe all over the place, women get married quickly. You know just when a lady is married, she forgets everything else. In rural areas, ladies just think of marriage while in urban areas, ladies are committed for studies.*” (Provincial Reproductive Health Coordination, Bunia)

Several solutions were proposed. Participants, mainly from the rural Adja and peri-urban Aru districts, addressed the problem of funding. They suggested a community-based sponsorship scheme, where a local committee establishes the criteria and processes to ensure that local people are supported financially to attend training and return to serve locally.

“*… they could encourage the local chief to send at least one person from the area to go to study midwifery. If in each health catchment area there is one person going to study midwifery, after 4 years, the problems of shortages can be solved, as they are children who grew up in the area*” (DHMT, Man, Adja)

Other participants addressed the problem of demand for training, proposing community awareness activities at churches, markets and over the radio, that encourage girls and young women to enrol in school and nursing school education.

“*So, it is better in that condition to make sure that they organise community awareness, even in churches to encourage parents to send their children in schools, and also promoting girls’ education*.” (DHMT, Man, Adja)

### Regulation

All participants in Adja and Aru and head nurses in Bunia proposed that the central government should prioritise civil service registration of midwives working in rural areas. The process for registration is very time consuming and it is only when midwives are registered that they are included on the payroll and receive salary. Some midwives have been working for 5 years and are waiting to be registered and one midwife in Adja district was reported to be working for more than 40 years without registration.

“*So, there is a real need for the government to register and pay health workers, with a special priority for those working in rural health facilities.*” (Church Medical Coordination)

### Financial incentives strategies

Participants from all districts explained that the best ways to improve attraction and retention of health workers in rural and remote areas is through making sure that they receive their salary, their rural and remote allowance, and a risk allowance.

“*I think that it is better for the government to pay all health workers, instead of being selective* [paying only those in urban health facilities]*, otherwise, those who are not paid* [in rural and remote areas] *will get discouraged and might decide to leave and that will not contribute to improve attraction, as no one would like to commit themselves to work knowing that they will not receive salary or risk allowances from the government*.” (DHMT, Man, Adja)

Meanwhile, financial constraint of the government was identified as the main challenge for implementation of this strategy, either because of insufficient budget allocated to health or financial planning for implementing strategies was not carefully thought through.

“*I think the major challenge could be weak implication of the government in dealing with some aspects where they are responsible, such as … making sure that financial motivation of health workers working in rural health facilities are better than that of urban areas. But also, we can consider a poor budget allocated to the health sector, just around 5% of the national budget. And the budget allocated for paying health workers are also very poor, especially for nurses and midwives*.” (Provincial Health Office, Man, Bunia)

According to participants from all three districts, there are some challenges that are difficult to overcome as they are the responsibility of the central government, i.e. improving financial initiatives.

“*At the national level, the government should respect and implement policies they have developed. If the government respected those policies elements, they would have made some changes by now*.” (Nursing School, Aru)

At the district and facility levels, participants proposed community mobilisation to use health services as that will increase income generation from user fees and, as a consequence, could be used to support health workers in the absence of salary or allowances.

In Adja district, providing land for cultivating food can help overcome financial challenges as described by one DHMT member:

“*… the local chief could give a piece of land so that the local population cultivates for the midwives working in the health facilities, or they can grow food for all health workers in the health facility as they do not benefit much from the health facilities.*” (DHMT, Man, Adja)

### Personal and professional support strategies

All participants called for the central government to provide more attractive living conditions, including housing that is close to the facility as well as safer and more supportive work environments.

“*I think it is important they improve living conditions of health workers, especially building houses for health workers in rural health districts. As health workers coming from urban areas were used to sleeping well in good conditions in their own houses, that is why they should improve their living conditions in relation to their housing.*” (Head Nurse, Man, Aru)

All participants from Adja district and nurses and midwives from Aru as well as some Provincial Health Office delegates insisted on improving working conditions through the provision of equipment and supplies, strengthening supportive supervision and in-service training to midwives as well as by promoting good leadership at the district and facility level.

“*Actually, as someone put it, rural health facilities need to be equipped with equipment and supplies, and that can also be a factor that will attract qualified midwives in rural areas.*” (Midwife, Woman, Adja)

The Provincial Health Office delegates described the need for increasing communication between urban and rural areas for both work and personal/family reasons by improving security in rural areas as well as improving road conditions.

“*Some more strategies to improve attraction in rural areas could be improving living conditions in rural areas, by ensuring security, and also making sure that road infrastructure conditions are improved, as that will facilitate communication between urban and rural areas.*” (Provincial Reproductive Health Coordination, Bunia)

Head nurses, midwives and DHMT delegates recognised that developing and maintaining good relationships within health facilities and with local communities are key to retention.

“*I think they should avoid conflicts in the area. Despite the poor salary, if the person works with good relationships with the local population and other health workers in the facility, the person can be happy to continue serving*” (Midwife, Man, Aru)

There were several challenges to implementing these strategies. For example, the central government’s role, as described by a member of the Provincial Health Office: “*I think the major challenge could be weak implication of the government in dealing with some aspects where they are responsible.*” (Provincial Health Office, Man, Bunia)

To overcome this challenge, participants from Aru and Adja proposed some local strategies, including lobbying NGOs to supply equipment to facilities; mobilising churches to improve their health facilities’ environments; encouraging local communities to build houses for health workers around facilities; and contributing user fees from the first delivery in the month to the district development fund, which can be used to improve the facility buildings and purchase equipment or supplies.

“*So, lobbying to NGOs intervening in the area so that they contribute with equipment in health facilities, especially for maternity services, that can contribute to retain health workers.*” (Midwife, Woman, Adja)

“*We told head nurses to take the first delivery of the month, and that delivery fee is put in the district development fund. So, we keep that money from each health facility in that development fund. Each health facility deposits per month 40,000 shillings* [US $11]*, they bring that money in the district health office, that money is for the development of our health facilities. At the end of the month, during monitoring meeting attended by all head nurses, head nurses bring that contribution for the month, and they decide to whom to serve that money, according to their health facilities development plan.*” (DHMT, Man, Adja)

However, even if other strategies are put in place, where insecurity and conflict continue, health workers will leave for safer areas:

“*But with these conflicts, and wars, many qualified midwives and other health workers decided to leave rural health facilities and rushed either here in Bunia or in other urban areas which appeared to be safer.*” (Provincial Health Office, Man, Bunia)

Overcoming insecurity in rural areas is seen as the responsibility of the provincial and central governments. However, participants proposed raising awareness amongst the community about the consequences of joining militia groups and creating activities to occupy the youth.

“*We should also include youth leaders. I think they should also create employment for young people as well as development project, covering different sectors, such as agriculture, training, mechanics, sewing, and so on. Of course, we will need NGOs to support those initiatives*” (DHMT, Bunia)

### Other local strategies

The participants came up with other strategies to attract and retain midwives to rural areas. First, head nurses could identify midwifery or nursing students and encourage them to come to their facilities for placement whilst they are in training, providing them with an enjoyable experience, so that they consider returning to work there.

“*I would also like to add that each head nurse could make efforts to identify those who study in nursing schools and colleges, those studying midwifery, at the end of the year, they should do their best to gain their confidence. I have managed to practice that, and some actually came.*” (Head Nurse, Man, Adja)

Second, most just-qualified midwives who are deployed to rural facilities are single. Marriage to local men would ensure that they stay in that area. It was suggested that facilities can organise social events so that young single people meet:

“*… you also know that, as we are talking of midwives, most of them are young girls, those who just complete their training. So, they are deployed in the area as single midwife. I would say that another strategy to retain them is that men from the area should make sure they take them for marriage. Young men need to make sure they ask them for marriage as they will contribute to serve their community.*” (DHMT, Man, Adja)

“*You know that recently there were two midwives from the referral hospital who left for marriage, because boys from the area did not make a step towards them, so other men from somewhere else came to ask them for marriage, and they had to leave.*” (DHMT, Man, Adja)

Third, participants from Aru and Adja districts proposed that the DHMTs integrate traditional birth attendants (TBAs) into the facilities so that they support midwives in providing services:

“*They should consider the importance of traditional birth attendants, by recruiting them and integrating them in the health facilities so that they work together with qualified midwives in maternity services, because they are very influential*.” (Midwife, Woman, Adja)

Workshop participants discussed several challenges with this strategy, which include limited resources for remuneration and TBAs may continue or even increase home deliveries as they may be perceived by the community as ‘midwives’. To overcome these challenges, TBAs could receive some of the income generated locally from user fees in the facility, like other health workers. Introducing fines to TBAs who do home deliveries may discourage this practice:

“*You know, in one of the areas here in Adja, the local authority has forbidden home delivery. If there is a case of home delivery, both the woman having delivered and the traditional birth attendant have to pay a fine of a goat each (30$).*” (Head Nurse, Man, Adja)

## Discussion

This study aimed to identify strategies, based on the research presented, that can help to attract, support and retain midwives in the fragile and rural Ituri province. Using the WHO framework on retention of health workers in remote and rural areas, workshop participants were able to reflect on the midwifery situation in their districts and province and develop strategies that were based on their local context, identifying and solving implementation challenges. The strategies selected are not new and have been well documented in the literature [11, 40–45]. However, they have been selected in a way that ensures that they are contextually relevant – a process that is not well covered in the literature. The findings go further to consider how, in a multi-stakeholder environment, the strategies can be implemented – again, an area not well covered in the literature. We now discuss three areas – the feasibility of these strategies; the need for collaboration between different levels of the health systems for effective implementation of the proposed strategies; and the collaborative approach in developing the strategies.

### Feasibility of strategies

With regard to education, two key strategies were proposed, namely including midwifery courses in nursing schools in rural areas and recruitment and training of students from rural areas. Including midwifery courses in rural nursing schools can have an impact on improving the attraction and retention of midwives in rural areas [40, 46], yet this may be challenging to implement in Democratic Republic of Congo, where only 32% of the schools of nursing are government run and the government may be reluctant to invest in schools of nursing and midwifery in rural areas [20]. Churches own the majority of health facilities in the Democratic Republic of Congo [20] and, therefore, creating church-funded nursing schools in remote areas where there are none or including midwifery in the existing nursing schools could be considered as an alternative viable solution. The recruitment and training of students with a rural background can be an effective strategy to improve the attraction and retention of health workers in rural areas [11, 40, 41] and this was an important element of conflict-affected Liberia’s workforce rebuilding strategy [42]. In eastern Democratic Republic of Congo, most graduates from rural medical schools worked in rural health facilities [46]. Involving the local community in the selection and support of students is a potentially powerful strategy; however, open and transparent processes for selection and dialogue and agreement with provincial district health authorities about the deployment of the students is crucial [43]. Central to this is the selection of women students as female midwives are more accepted in rural areas; this requires cross sector collaboration and long-term support so that girls, their families and their communities are encouraged to support their schooling, delay marriage, and focus on their education and career [47].

Providing a regular salary and allowances was an important strategy raised by the workshop participants; this has been shown as being significant in fragile settings [48, 49]. It is critical that midwives are able to support themselves and their families whilst continuing to serve their communities [11, 41, 44, 50, 51]. However, it is clear from this study and others that very few health workers in Ituri province receive salary or allowances, and this is particularly the case for those working in rural areas [33]. Ituri province may well be specifically disadvantaged as it is estimated that, in the whole country, 33% of health workers receive a salary from the government and ~65% receive their risk allowances [18, 20, 52]; the majority of these health workers are based in urban areas [52]. This is one of the reasons why health workers remain in urban areas. Therefore, in rural contexts, health workers rely on income generated locally from user fees [18]. Workshop participants proposed that local income generation should be continued and strengthened. However, this raises important moral issues – most communities in rural Democratic Republic of Congo are poor and asking them to support health workers will place additional burdens on them and push them into more poverty, promoting a situation of inverse equity, whereby those with the most need are expected to pay for services. There are many who will not be able to access services because of these fees.

In a context where the government is limited in providing professional and personal support because of financial constraints and insecurity, the participants came up with strategies that are both resilient and embedded in the local context. These include lobbying local NGOs to support facilities, as experienced in rural South Africa and Sudan [53, 54]; organising district level strategies, such as the district development fund initiated in Adja district, which is used to improve facilities and purchase basic equipment and supplies; rallying churches to improve living and working conditions in their facilities; actively approaching students to work in their areas; and mobilising communities to improve living conditions by building houses at the facilities, to support local marriage of young single midwives, and to discourage youth enrolment in local militia. There is no one single ‘magic bullet’ to solve the problem of attraction and retention of midwives but, rather, a combination of different strategies and at different levels of the health system is needed.

### Need for collaboration between different levels of the health system for effective implementation of the proposed strategies

There is a range of stakeholders responsible for the implementation of the proposed attraction and retention strategies, i.e. central government, the Provincial Health Office, the DHMT, health facilities, community members and others such as churches, NGOs and nursing schools. Supporting a clear collaboration mechanism between all stakeholders will ensure a better outcome of more midwives working in rural areas [55]. For instance, recruiting and training rural background students through a community-based sponsorship scheme requires collaboration between the sponsorship scheme managing committee, who select and send candidates for midwifery training, and the Provincial Health Office and the DHMT, who are responsible for deployment [20, 21]. Without sustained collaboration, community selected and supported students, once graduated, may be deployed elsewhere, creating tension between the different groups and community mistrust of the scheme, which will limit its sustainability. Good collaboration and systems and processes that build trust and principles of reciprocity and mutuality between these health systems actors can help promote positive outcomes [56–59] such as a more informed recruitment and deployment system.

### Collaborative approach to developing strategies

This study used an innovative approach to developing the strategies, bringing together a range of stakeholders who are involved in human resource management, service delivery and midwifery, and working with researchers to co-produce knowledge and strategies. The approach includes presenting relevant data from routine information systems and research to help ground the discussion in reality. The availability of staffing data is a major strength of the region, as it is often lacking in fragile and conflict-affected contexts [49] and in other parts of Democratic Republic of Congo [60]. Thus, the current study used reflection on locally generated evidence and policies and the rich and detailed knowledge of the local contexts to develop feasible and potentially effective strategies. This is a learning health system in action – stepping back, taking stock of what is happening and why, reflecting on evidence and real-world experience, debating and discussing options, and coming up with solutions. Such an approach can embed learning into all aspects of decision-making in health systems [61]. It was facilitated by a research team knowledgeable about the topic and embedded in the context, with strong and trusting relationships with the participants built over several years. Local credibility, a good knowledge of the context and trust are key components needed for the co-production of knowledge [59, 62] and generally lacking in fragile settings.

### Implementing change

In uncertain environments, managers at the local level need the flexibility and skills in human resource management to effectively implement changes [63, 64]. The engagement of managers in the workshops show that they are committed to change, though they should be supported somehow. Secondly, such suggestions for changes made in the workshops may well be accepted in principle, but so often organisational inertia prevents them from actually being implemented until an appropriate ‘window of opportunity’ arises. Such a window became available in Sierra Leone in 2010, whilst it was still recovering from conflict, with the introduction of the Free Health Care Initiative [65]. In the Democratic Republic of Congo, the aftermath of Ebola and COVID-19 may possibly provide the window of opportunity needed in Ituri Province and elsewhere.

### Strengths and limitations

The strength of this study is based on the participatory research methodology used, where different health systems actors, with a good understanding of the context, came together and reflected on possible solutions in relation to the attraction and retention of midwives in rural and remote fragile Ituri Province. Workshops were conducted by a Democratic Republic of Congo team embedded within the context of fragile rural Ituri. However, there were limitations. One of the limitations is that we only included midwives who have worked in rural areas. The inclusion of midwifery students in their final year, midwives newly recruited and deployed in urban health facilities as well as midwives working in private for profit facilities, would provide views about what strategies would encourage them to opt for rural employment.

## Conclusion

Rural Democratic Republic of Congo is severely affected by shortages of health workers, especially midwives. There is an urgent need for contextual strategies to improve the attraction and retention of rural midwives. Through a participatory approach using a workshop methodology, health systems stakeholders developed potential strategies on education, professional and personal support, and finances to attract and retain midwives. These strategies were based on local evidence, their rich and detailed knowledge and experience, and their reflections on the unique context of the Democratic Republic of Congo. This participatory approach can promote learning health systems and develop pragmatic strategies to attract and retain health workers in fragile, remote and rural settings.

Box 1 Midwifery in the Democratic Republic of Congo

**Table:** 

- The midwifery profession, as defined by the International Confederation of Midwives (ICM) is new in DRC (since 2013) [66]. There is no legal regulatory structure to uphold the midwifery profession. It is therefore not possible to ensure a high-quality workforce of midwives in DRC [67]. - The midwifery association was established in 2000, is well connected and accepted, with 1700 members and a member of ICM, but needs more resources to function effectively. - Midwifery education is managed by two different government ministries: Public Health and Higher Education	
**Ministry of Public Health (MoPH)**	**Ministry of Higher Education (MoHEd)**
** 1. Midwifery training** *A3 midwives: Since the colonial period until late 1980s, midwifery training began at a secondary-school level (Institut de Techniques Médicales: ITM)* [67] - Entry requirements: 10 years of education (6 years of primary and 4 years of secondary) - 2 years of midwifery in nursing schools training A3 level birth attendants (accoucheuses A3) - The A3 midwifery programme was abolished and replaced by a four-year midwifery education programme (A2).	**1. Midwifery training** *A1 midwives (undergraduate degree) (Institut Supérieur de Techniques Médicales : ISTM)* [67] - Entry requirements: A2 midwives or 12 years of education (6 years in primary and 6 years in secondary schools) - 3 years of midwifery in nursing colleges training A1. Since 2013, when the country’s educational reform took place, a three-year midwifery education (sage femme) is conducted at a higher education level, which is in line with midwifery international norms and standards
*A2 midwives (diploma level) (Institut Techniques Médicales : ITM)* - Entry requirements: 10 years of education (6 years of primary and 4 years in secondary) - 4 years of midwifery in nursing schools	*A0 midwives (Post graduate degree) (at Institut Supérieur de Techniques Médicales : ISTM)* - A1 midwives - 2-year post graduate training programme in obstetrics and gynecology (A0) in a few nursing colleges
**2. Types of training providers** - The government - Faith Based Organizations (Not-for-profit private sector)	**2. Types of training providers** - The government - Faith Based Organizations (Not-for-profit private sector)
**3. Location** - Urban areas (in a few schools only) Midwifery programme not organized in most schools) - Rural areas (in a few schools only)midwifery programme not organized in most schools)	**3. Location** - Urban areas (nursing colleges: ISTM, concentrated in urban areas) - Rural areas

Box 2 Availability and distribution of skilled birth attendants in Ituri: key findings [33]

**Table:** 

• The shortages of midwives are the most extreme, especially in peri- urban (24.9% of posts filled) and rural districts (7.2% of posts filled), while there is a surplus of doctors and nurses in urban and peri-urban districts (> 100%) • While the number of doctors and nurses has increased in urban, peri-urban and rural districts from 2013 to 2017, the number of midwives has decreased in peri-urban and rural districts • There is clear gender and occupational segregation: doctors and nurses are more likely to be men, whereas midwives are more likely to be women; there are more women nurses in the urban district • The projections of training outputs show a surplus of doctors and nursing increasing, whilst the shortfall for midwives remains above 75%

Box 3 Midwives work experiences in Ituri Province: key findings [34]

**Table:** 

• Midwives joined midwifery for different reasons, including a wish to solve problems, fulfilling childhood aspirations and wanting to be role models for their community • Midwives faced health systems-related challenges, including severe shortage of qualified co-workers, poor working conditions due to lack of equipment, supplies and professional support, and no salary from the government, apart from risk allowances received by some • Midwives also experienced socio-cultural challenges: gender norms ofmale midwives not being accepted in rural communities (most male midwives work in urban areas), married female midwives not being allowed to work due to family responsibilities, women attending antenatal services late in pregnancy or coming to the facility on their own for delivery, and a culture of blame when there are deaths or complications • Midwives have developed coping strategies such as generating income and food from farm work, lobbying local organisations for supplies and training traditional birth attendants to work in facilities

Box 4 Policies on attraction and retention of health workers in rural and remote areas in the Democratic Republic of Congo [20, 21]

**Table:** 

**1. Education** - Integrating midwifery and other courses in nursing schools **2. Regulatory** - Registering eligible health workers and including them on payroll quickly so that they receive salary and allowances - Applying a standardised pay rate to health workers having the same qualification both in urban and rural areas - Equitable initial deployment of health workers in health facilities according to needs and redeployment of surplus health workers **3. Financial incentives** - Ensuring regular payment of salary and allowances - Implementing rural placement allowances **4. Personal and professional support** - Improving working conditions of health workers in rural areas by supplying equipment and supplies, providing supportive supervision and in-service training - Construction of staff houses at facilities **5. Others** - Development and sensitisation of HRH staffing standards - Control of the deployment of registered health workers between facilities - Strengthening the HRH information system - Organising’ payment sites in rural areas close to health workplaces

## Data Availability

Data are available from the corresponding author on reasonable request.
